# Eligibility and discontinuation of prenatal care in a freestanding birth center: a cross-sectional study

**DOI:** 10.1590/0034-7167-2024-0015

**Published:** 2025-09-26

**Authors:** Franciely Schermak, Maria Luiza Gonzalez Riesco

**Affiliations:** I Universidade de São Paulo. São Paulo, São Paulo, Brazil

**Keywords:** Birthing Centers, Pregnancy, Prenatal Care, Midwifery, Cross-Sectional Studies, Centros de Asistencia al Embarazo y al Parto, Embarazo, Atención Prenatal, Enfermería Obstétrica, Estudios Transversales

## Abstract

**Objectives:**

to analyze the factors related to eligibility and discontinuation of prenatal care in a freestanding birth center (FBC).

**Methods:**

a cross-sectional study, conducted at the Casa Angela FBC, SP, Brazil, involving 9,954 women registered between 2020-2022. Descriptive analysis was performed, including odds ratios.

**Results:**

43.6% were eligible for prenatal care and 62.9% had their care discontinued. A higher level of education, higher income, Asian/Indigenous ethnicity, and living with a partner increased the chance of eligibility; older maternal age, a higher number of pregnancies, brown/black skin, and private health insurance decreased this chance. Brown/black skin and Indigenous ethnicity increased the chance of prenatal care discontinuation; older maternal age, a higher number of pregnancies, higher education, higher income, Asian ethnicity, living with a partner, and private health insurance decreased this chance.

**Conclusions:**

sociodemographic factors and clinical and obstetric history influence both the ineligibility and discontinuation of prenatal care in CPN.

## RESUMEN

**Objetivos:**

analizar los factores relacionados con la elegibilidad e interrupción del seguimiento prenatal en un centro de parto normal peri-hospitalario (CPNp).

**Métodos:**

estudio transversal realizado en el CPNp Casa Angela, SP, Brasil, con 9.954 mujeres registradas entre 2020-2022. Se realizaron análisis descriptivo y cálculo de la razón de probabilidades.

**Resultados:**

el 43,6% eran elegibles para el cuidado prenatal y el 62,9% tuvieron una interrupción del seguimiento. Mayor nivel educativo, ingresos elevados, etnia asiática/indígena y vivir con pareja aumentaron la probabilidad de elegibilidad; mayor edad, número de embarazos, color de piel parda/negra y seguro de salud disminuyeron esta probabilidad. Color de piel parda/negra y etnia indígena aumentaron la probabilidad de interrupción del cuidado prenatal; mayor edad, número de embarazos, mayor nivel educativo, ingresos elevados, etnia asiática, vivir con pareja y seguro de salud disminuyeron esta probabilidad.

**Conclusiones:**

factores sociodemográficos e historial clínico y obstétrico influyen en la inelegibilidad y en la interrupción del seguimiento prenatal en CPNp.

## INTRODUCTION

Freestanding birth center (FBC) and alongside birth center (ABC) birth centers are strategies designed to provide humanized care based on scientific evidence and contribute to the reduction of cesarean rates^(1)^. Since 1999, the Brazilian Ministry of Health (MH) has established specific legislation and guidelines for the creation and operation of FBC^(2)^. According to Ordinance GM No. 11/2015, a FBC must be located near a reference hospital, at a distance that can be covered in less than 20 minutes with appropriate transport. The center must ensure the transfer of the woman and the newborn (NB) to the hospital in cases of risk or complications, 24 hours a day, seven days a week; it must also provide support services as a reference and ensure that the woman and the NB remain in the PPP room (pre-delivery, delivery, and post-delivery) from admission until discharge^(1)^.

Also referred to as a “birth center”, it offers a “social model of care”. In contrast to the typical hospital model, the FBC recognizes childbirth as an important life transition for women and families, tied to positive experiences in the context of motherhood and fatherhood. It features an environment and professional team that are effective in reducing interventions and promoting the mental and social well-being of its users^(3)^.

This healthcare facility should be integrated into the primary care network, serving women in its coverage area. It provides care for normal deliveries and is intended for low-risk women. Obstetric nurses and midwives are responsible for comprehensive care, with support from other nursing professionals, and may include a multidisciplinary team^(1)^.

During prenatal care, pregnant women should be informed about the options for the place of delivery and, if they meet the criteria for low risk, they have the right to choose where they wish to give birth, receiving quality information and assistance. According to the national guidelines for normal birth care from the MS, “Low-risk pregnant women who opt for delivery in a Birth Center (extra, peri, or intra-hospital), if available in their coverage area or nearby, should be supported in their decision”^(4)^.

Pregnancy is a physiological phenomenon and should be considered by pregnant women and healthcare teams as part of a healthy life experience, involving physical, social, and emotional changes. However, some pregnant women may present a higher likelihood of unfavorable outcomes due to risk factors; in such cases, they are classified as “high-risk pregnant women”. When maternal and perinatal morbidity and mortality rates are equal to or lower than those of the general population, pregnancy is considered low risk, without the need for high-density health technology^(4)^.

Gestational risk factors can be promptly identified during prenatal care through anamnesis, general and obstetric physical exams, and complementary tests. The presence of one or more of these factors does not imply the immediate need for more advanced diagnostic resources than those commonly offered in low-risk prenatal care. However, these factors indicate that the healthcare team should exercise increased vigilance with these pregnant women. When factors associated with a worse prognosis are identified, the use of higher-density technological procedures, such as specialized laboratory or imaging tests, hospital admission, and fetal malformation monitoring, may be required^(4)^.

According to the literature, pregnant women do not always need to be referred to high-risk prenatal care; many situations can be resolved within primary healthcare services, and real risk cases or avoidable situations should be referred. The pregnant woman may return to primary care once the situation is resolved. Around 10% to 15% of pregnant women require high-risk follow-up^(5)^.

Risk factors are classified into three main groups that must be identified and managed, with indications for referral to high-risk care or emergency services. According to MS guidelines, these factors are classified as: 1) Risk factors that should be identified and managed (related to individual characteristics, unfavorable sociodemographic conditions, previous reproductive history, and current pregnancy); 2) Risk factors that indicate the need for referral to high-risk care (related to pre-existing conditions, previous reproductive history, and current pregnancy); 3) Factors that indicate referral to emergency services^(5)^.

In 2018, in the United Kingdom, an initiative by the Midwifery Unit Network (MUNet) and the European Midwives Association (EMA) resulted in the publication of the *Midwifery Unit Standard*, which provides guidelines for eligibility and the choice of the place of birth, based on scientific evidence^(6)^.

In Brazil, despite existing policies, the FBC model is still little known and rarely available. In 2022, only 0.7% of births occurred in non-hospital healthcare settings^(7)^. On the other hand, there is a growing demand from women seeking a humanized birth outside the hospital context, as observed in the service analyzed in this study. Moreover, as these environments are designed for low-risk women, there is a high number of transfers to hospitals^(8,9)^, and it is also presumed that the number of interruptions in care during pregnancy is significant.

In 2019, the São Paulo Municipal Health Department established actions to promote the humanization of care and improve the quality of childbirth and delivery services. Among these actions, a working group was created to develop the *Technical Manual for Birth Centers*, with the objective of systematizing the work processes and aligning the technical demands and needs of parturients and their NB in the two birth centers operating in the city. The manual establishes criteria for monitoring the pregnancies of women cared for in this setting^(10)^.

According to the manual, pregnancy monitoring in a FBC is optional, but upon admission for delivery, it must be verified that the pregnancy is low-risk, without factors indicating maternal or neonatal morbidity. A thorough evaluation must be conducted to ensure that the delivery is also low-risk^(10)^.

The present study was proposed considering access barriers, the lack of recognition regarding the safety and functioning of FBCs, and the limited literature on the topic.

## OBJECTIVES

To analyze the factors related to the eligibility and discontinuation of prenatal care for women in a FBC.

## METHODS

### Ethical aspects

The study was conducted in accordance with national and international ethical guidelines and approved by the Research Ethics Committee of the School of Nursing, University of São Paulo, whose approval is attached to this submission. The use of an informed consent form was not required, as this study utilized the institution’s database and electronic health record system reports, with the data analyzed and presented in an aggregated manner.

### Study design, period, and location

This is a cross-sectional study using data from 2020 to 2022. The data sources included the registry of pregnant women, electronic medical records, monthly reports sent to the São Paulo Municipal Health Department, and the birth database. The reporting of this manuscript follows the *Strengthening the Reporting of Observational Studies in Epidemiology* (STROBE) guidelines.

The study was conducted at the Casa Angela FBC - Center for Humanized Birth, located in São Paulo, Brazil. It is a non-profit institution managed by the Monte Azul Community Association, serving users of the Unified Health System (SUS in Portuguese). Only low-risk pregnant women are admitted for delivery.

Pregnant women, at any stage of pregnancy, register online, providing identification, sociodemographic, obstetric, and clinical data, along with the main reasons for ineligibility (previous cesarean delivery, diabetes, use of medication for hypertension, curettage up to six months before the current pregnancy, multiple pregnancies). After this step, the pregnant woman undergoes an online screening consultation. If eligibility is confirmed, the first in-person prenatal consultation is scheduled, starting at the 28th week of pregnancy.

Prenatal care and deliveries are assisted by obstetric nurses and midwives. When there is a risk factor that prevents the woman from giving birth at the FBC, prenatal care is discontinued, and the pregnant woman is informed of the reason and referred to the primary health unit (PHU) where she is receiving prenatal care. The integration with the municipal network enables the formalization of the referral and counter-referral flows with PHU. In cases of emergency, the pregnant woman is referred to the reference hospital or the hospital of her choice, if she has private health insurance.

Gestational follow-up occurs through individual and group consultations (held with women at the same gestational age), alternating with PHU consultations, following the periodicity recommended by the MH. In every prenatal consultation, risk factors are reassessed, and if at any point a complication arises that prevents the delivery from taking place at the FBC, the care is discontinued.

Admission may occur from 37 weeks of gestation up to 41 weeks and 0/7 days. When maternal or neonatal transfer to the hospital is necessary, it is carried out by the institution’s ambulance to the reference hospital, located near the FBC.

### Population and inclusion/exclusion criteria

The study population consisted of all pregnant women who registered for prenatal care and delivery during the period from January 1, 2020, to December 31, 2022. The inclusion criterion was the completion of the registration process to begin follow-up, regardless of whether prenatal care at the FBC had been initiated or not. Women who completed the registration but for whom there was no information regarding eligibility or prenatal care follow-up in the FBC database were excluded.

### Exposure and outcomes

The exposures considered were: maternal age, number of pregnancies, education level, household income, skin color/ethnicity, cohabitation with a partner, and health insurance. The outcomes were eligibility for prenatal follow-up at Casa Angela and the discontinuation of prenatal care among women who were not admitted for delivery at the FBC.

### Data analysis and statistics

The data were organized and categorized in Google Sheets, imported into Excel, and analyzed using R software, version 4.3.2.

For descriptive analysis of continuous variables, the mean, standard deviation (SD), median, minimum and maximum values, lower quartile (Q1), and upper quartile (Q3) were calculated. For categorical variables, absolute and relative frequencies were calculated.

To analyze the relationship between exposure variables and the outcomes of eligibility, discontinuation, and admission for delivery, the odds ratio (OR) was calculated.

## RESULTS

From January 2020 to December 2022, 10,958 women registered to begin prenatal care and delivery at the FBC. Of these, 1,004 (9.2%) were excluded because information regarding eligibility and follow-up at Casa Angela was not available in the data sources used for the study.

**Figure 1:**
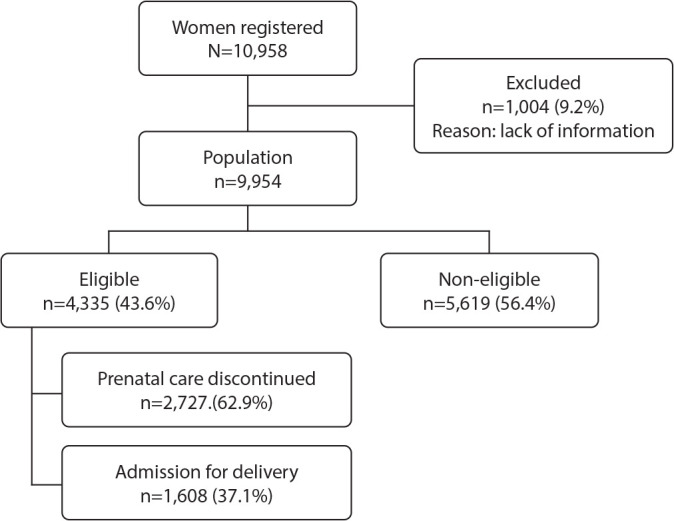
Flowchart of registered and attended women, São Paulo, São Paulo, Brazil, 2020-2022

The study population consisted of 9,954 women who completed registration with available information regarding eligibility for care at the FBC. According to the online screening criteria, 5,619 (56.4%) did not meet the protocol requirements for entering prenatal care, while 4,335 (43.6%) were considered eligible for follow-up at Casa Angela. Among the eligible women, 1,608 were admitted for delivery, and 2,727 had their care discontinued, resulting in a discontinuation rate (failure rate) of 62.9% (Figure 1).

Tables 1 and 2 show the sociodemographic and obstetric characteristics of the population.

**Table 1:** Distribution of eligible women and those with prenatal care discontinuation according to sociodemographic characteristics, São Paulo, São Paulo, Brazil, 2020-2022

Variable	Eligible		Total	Prenatal care discontinuation	Admitted for delivery	Total
	Yes	No				
	n=4,335 (%)	n=5,619 (%)	N=9,954 (%)	n=2,727	n=1,608	N=4,335
Skin color/ethnicity	4,112	4,861	8,973	2,645	1,467	4,112
White	1,923 (46.7)	2,191 (53.2)	4,113 (45.8)	1,185 (61.6)	738 (38.4)	1,923 (46.9)
Brown	1,469 (44.9)	1,804 (55.1)	3,273 (36.5)	975 (66.4)	494 (33.6)	1,469 (35.7)
Black	606 (44.9)	745 (55.1)	1,351 (15.1)	413 (68.2)	193 (31.8)	606 (14.7)
Asian	84 (46.9)	95 (53.1)	179 (2.0)	51 (60.7)	33 (39.3)	84 (2.0)
Indigenous	30 (52.6)	27 (47.4)	57 (0.6)	21 (70.0)	9 (30.0)	30 (0.7)
Education level	4,109	4,862	8,971	2,643	1,466	4,109
Incomplete elementary	130 (35.8)	233 (64.2)	363 (4.0)	93 (71.5)	37 (28.5)	130 (3.2)
Complete elementary	192 (36.0)	342 (64.0)	534 (5.9)	135 (70.3)	57 (29.7)	192 (4.6)
Complete high school	2,193 (45.0)	2,686 (55.0)	4,879 (54.4)	1,423 (64.9)	770 (35.1)	2,193 (53.4)
Complete higher education	1,594 (49.9)	1,601 (50.1)	3,195 (35.6)	992 (62.2)	602 (37.8)	1,594 (38.8)
Lives with a partner	4,213	5,542	9,755	2,718	1,495	4,213
Yes	3,506 (46.2)	4,076 (53.8)	7,582 (77.7)	2,221 (63.3)	1,285 (36.7)	3,506 (83.2)
No	707 (32.5)	1,466 (67.5)	2,173 (22.3)	497 (70.3)	210 (29.7)	707 (16.8)
Household income (minimum wages)	4,112	4,865	8,977	2,645	1,467	4,112
≤ 1	642 (39.7)	976 (60.3)	1,618 (18.0)	440 (68.5)	202 (31.5)	642 (15.6)
2	1,352 (46.8)	1,540 (53.2)	2,892 (32.2)	855 (63.4)	494 (36.6)	1,352 (33.0)
3	898 (47.6)	989 (52.4)	1,887 (21.0)	594 (66.1)	304 (33.9)	898 (21.8)
4	480 (48.3)	512 (57.7)	992 (11.0)	288 (59.9)	192 (40.1)	480 (11.6)
5	302 (52.2)	275 (47.8)	577 (6.4)	190 (62.8)	112 (37.2)	302 (7.3)
6	155 (40.4)	229 (59.6)	384 (4.3)	101 (65.2)	54 (34.8)	155 (3.8)
> 6	283 (45.1)	344 (54.9)	627 (7.0)	174 (61.5)	109 (38.5)	283 (6.9)
Health insurance	4,117	5,063	9,180	2,646	1,471	4,117
Yes	538 (36.2)	948 (63.8)	1,486 (16.2)	307 (57.1)	231 (42.9)	538 (13.1)
No	3,579 (46.5)	4,115 (53.5)	7,694 (83.8)	2,339 (65.4)	1,240 (34.6)	3,579 (86.9)

**Table 2:** Distribution of eligible women and those with prenatal care discontinuation according to age and number of pregnancies, São Paulo, São Paulo, Brazil, 2020-2022

Variable	n	Mean (SD)	Median	Min-Max.	Q1-Q3
Eligibility					
Age (years)					
Eligible	4,332	27.3 (5.4)	27	13-44	23-31
Not eligible	5,611	27.4 (5.8)	27	14-45	23-31
Number of pregnancies					
Eligible	4,241	1.5 (0.9)	1	1-8	1-2
Not eligible	5,585	1.6 (0.9)	1	1-8	1-2
Prenatal care discontinuation					
Age (years)					
Prenatal care discontinuation	2,724	27.3 (5.5)	27	13-44	23-31
Admitted for delivery	1,608	27.4 (5.2)	27	14-42	24-31
Number of pregnancies					
Prenatal care discontinuation	2,642	1.5 (0.8)	1	1-8	1-2
Admitted for delivery	1,599	1.6 (0.9)	1	1-8	1-2

The profile of the women, based on eligibility to start prenatal care and the discontinuation of follow-up, indicates that those under 20 years of age and those aged 40 and above were more prevalent among the non-eligible and those who experienced discontinuation. The mean, median, and age extremes were similar between eligible and non-eligible women and between those with and without discontinuation.

Regarding skin color or ethnicity, more than half identified as brown or black, with a higher prevalence among the non-eligible and those whose prenatal care was discontinued.

Most of the women had completed high school, followed by those with higher education; among the non-eligible women, those with lower education levels (without high school education) were more prevalent. However, among women with lower education (with incomplete or complete elementary education), the proportion of prenatal care discontinuation was higher compared to those with higher education.

In terms of relationships, more than ¾ of the women lived with a partner, and the vast majority reported not having health insurance. The proportion of eligible women was higher among those living with a partner, as was the proportion among those who did not experience discontinuation of prenatal care and were admitted for delivery. Among the eligible women, the proportion of those without health insurance was higher, in contrast to those admitted for delivery, where women with health insurance were more prevalent.

Regarding paid employment and household income, among the eligible women, those without paid employment, a monthly income of five minimum wages, and income ≥ four minimum wages were more prevalent. Among the women whose prenatal care was discontinued, those with paid employment, a monthly income ≤ one minimum wage, and income ≤ three minimum wages were more prevalent.

As for how women learned about Casa Angela, most were informed through friends or family, and only a small proportion learned from a healthcare professional. It is also noteworthy that the prevalence of non-eligible women who learned about Casa Angela through social media coincided with those who had their prenatal care discontinued.

Of the total number of women who had previously been followed at Casa Angela, the majority were eligible again, and among those whose prenatal care was discontinued, the proportion was higher if the woman had not had a previous pregnancy followed at Casa. Both eligibility and prenatal care discontinuation were more frequent among primigravidas. The central tendency and dispersion values for the number of pregnancies were also similar between eligible and non-eligible women, as well as between those whose prenatal care was or was not discontinued.

Regarding gestational age at registration, the groups were predominantly in the second trimester of pregnancy, with a lower mean, median, and minimum age among the non-eligible women and a higher age among those admitted for delivery. Of the 2,727 women whose prenatal care was discontinued, 242 (8.9%) had a gestational age ≥ 37 weeks (data not shown in the table).

Of the total number of non-eligible women and those whose prenatal care was discontinued, the reasons for non-eligibility and discontinuation were recorded in the database for only 1,363 (24.3%) and 1,466 (53.8%) women, respectively. For some women, there were up to two reasons for non-eligibility and up to three reasons for prenatal care discontinuation.

The main reason for non-eligibility for prenatal care at Casa Angela was a previous cesarean delivery, representing 37.6% of the reasons, followed by gestational diabetes mellitus (GDM) (11.0%) and gestational hypertensive syndrome (GHS) (9.8%). In turn, the main reason for prenatal care discontinuation was GHS, representing 15.6% of the reasons, followed by a positive result for Group B Streptococcus (GBS) screening in the vaginal or anal canal, and post-term pregnancy (data not shown in tables).

The reasons for non-eligibility and discontinuation were regrouped and classified, as presented in Table 3. Among the reasons for non-eligibility, those related to previous reproductive history were the most frequent, followed by current clinical complications and pre-existing clinical conditions before pregnancy. Regarding discontinuation, the most frequent reasons were related to current obstetric and clinical complications.

**Table 3:** Distribution of Women According to the Reasons for Non-Eligibility and Prenatal Care Discontinuation, São Paulo, São Paulo, Brazil, 2020-2022

Variable	n	%
Reason for non-eligibility	1,534	100
Previous reproductive history (cesarean or curettage)	614	39.9
Current clinical complication^*^	487	31.8
Pre-existing clinical conditions before current pregnancy†	196	12.8
Current obstetric complication‡	99	6.5
Current individual and sociodemographic conditions§	90	5.9
Other¶	48	3.1
Reason for prenatal care discontinuation	1,593	100
Current obstetric complication‡	826	51.8
Current clinical complication^*^	628	39.4
Current individual and sociodemographic conditions§	75	4.7
Pre-existing clinical conditions before current pregnancy†	52	3.3
Other¶	12	0.8

* *More frequent (>1%): gestational hypertensive syndrome or hypertensive peak, positive Group B Streptococcus, gestational diabetes mellitus, urinary tract infection, active or treated syphilis during pregnancy, anemia; †More frequent (>1%): fibroids, grade 3 obesity, thyroid diseases, asthma, anxiety disorder, and depression; ‡More frequent (>1%): post-term pregnancy >41 weeks, extreme fetal percentile, changes in amniotic fluid volume, abnormal cardiotocography, fetal ultrasound or Doppler, breech presentation, preterm labor, meconium, premature rupture of membranes, increased uterine height, intrauterine growth restriction, multiple pregnancy; ¶Situations that occurred only once or the reason was unclear in the professionals' notes.*

Table 4 presents the odds ratios (OR) for eligibility and discontinuation of prenatal care at Casa Angela, calculated for the exposure variables. The results for eligibility are as follows: each year of maternal age decreases the chance by 0.3%; each pregnancy decreases the chance by 15.6%; each level of education increases the chance by 10%; each income level increases the chance by 3%; compared to white women, brown women have a 7.3% lower chance, black women have a 7.4% lower chance, Asian women have a 0.7% higher chance, and Indigenous women have a 26.5% higher chance; living with a partner increases the chance by 78.4%; having health insurance decreases the chance of eligibility by 34.7%. The results for prenatal care discontinuation are as follows: each year of maternal age decreases the chance by 0.5%; each pregnancy decreases the chance by 10.0%; each level of education decreases the chance by 7.5%; each income level decreases the chance by 3.7%; compared to white women, brown women have a 22.9% higher chance, black women have a 33.3% higher chance; Asian women have a 3.7% lower chance, and Indigenous women have a 45.3% higher chance; living with a partner decreases the chance by 27.0%; having health insurance decreases the chance by 29.5%.

**Table 4:** Odds ratio (OR) for eligibility for follow-up and prenatal care discontinuation, São Paulo, São Paulo, Brazil, 2020-2022

Outcome	OR
Eligibility	
Maternal age	0.997
Number of pregnancies	0.844
Education level	1.100
Household income	1.030
Skin color/ethnicity	
White	1.000
Brown	0.927
Black	0.926
Asian	1.007
Indigenous	1.265
Lives with a partner	1.784
Health insurance	0.653
Prenatal care discontinuation	
Maternal age	0.995
Number of pregnancies	0.900
Education level	0.925
Household income	0.963
Skin color/ethnicity	
White	1.000
Brown	1.229
Black	1.333
Asian	0.963
Indigenous	1.453
Lives with a partner	0.730
Health insurance	0.705

## DISCUSSION

Although they may represent access restrictions, eligibility criteria for monitoring pregnant women are measures designed to minimize the risks of unfavorable maternal and neonatal outcomes, enabling the functioning of FBC. The impact of these criteria on prenatal screening and the admission of women for delivery is reflected in the failure rates, as more than half of the pregnant women seeking care at the FBC were not eligible, either due to pre-existing conditions or complications during pregnancy.

There are risk factors associated with pregnancy that, initially, do not justify referring the pregnant woman for high-risk, urgent, or emergency care^(5)^. Proper prenatal and postpartum follow-up requires continuous care, which facilitates the identification of these factors. The FBC, integrated into primary care, promotes the integration of referral and counter-referral services for the care of pregnant women, postpartum women, and NB.

Casa Angela conducts prenatal follow-up in partnership with PHU to maintain a connection with the primary care network, which also encourages pregnant women and network professionals to learn about the FBC care model. It is worth noting that prenatal consultations conducted at Casa Angela are not recorded in municipal information systems. Maintaining consultations at PHU also enables primary care to meet the minimum consultation indicators.

Factors related to individual characteristics and unfavorable sociodemographic conditions include extreme age. Adolescent pregnant women are more likely to develop complications such as GHS and preterm birth^(5,11-13)^. At Casa Angela, adolescent pregnant women are welcomed into a project dedicated to this age group, which includes starting prenatal care at any time before 28 weeks (different from the protocol for starting follow-up for other pregnant women). Even if the pregnant woman presents risk factors, prenatal care is maintained at Casa Angela, but the delivery is performed at the reference hospital.

The most common complications in late pregnancy are GDM, GHS, and premature rupture of membranes^(14)^. The literature shows that over 70% of pregnant women aged ≥35 years experienced some form of complication^(14)^, and more than half of the women in this age group had a cesarean section^(15)^. However, maternal age should not be considered an isolated factor for clinical and obstetric complications; factors related to past and current health and obstetric history, as well as socioeconomic conditions, should also be considered^(14)^. These studies partially corroborate the results of this study, in which each additional year of maternal age decreased the chance of eligibility and increased the likelihood of prenatal care discontinuation.

At Casa Angela, more than half of the women identified as brown or black, similar to data from the Live Birth Information System (SINASC in Portuguese) in the Southeast region in 2021^(16)^. Brown and black women face greater difficulties in accessing information and quality care, experience more obstetric violence, and account for 60% of maternal deaths^(17)^. The bias related to skin color and ethnicity is consistent with the results of this study, in which brown and black women, compared to white women, had a lower chance of being eligible for follow-up and a higher chance of prenatal care discontinuation.

Women in situations of socioeconomic vulnerability - those with lower education and low income - face challenges in accessing obstetric care, increasing the risk of avoidable obstetric complications due to the low quality of prenatal care, limited access to tests and other technologies, lack of knowledge, and greater exposure to violence^(18)^.

Access to and the quality of care during pregnancy can be affected by low education and income^(19)^. The profile of FBC users revealed a high level of education (the vast majority had completed high school or higher education), in contrast to the SINASC data. In the Southeast region and in Brazil in 2022, women with a high school or higher education represented 26.7% and 23.2%, respectively^(20)^.

Lower education and income were predominant among non-eligible women and those with discontinued prenatal care. Higher education increased the chance of eligibility and reduced the chance of prenatal care discontinuation. In turn, the chance of eligibility was directly proportional to higher income, while the chance of discontinuation was inversely proportional to higher income.

Individualized care during prenatal follow-up at the FBC can promote quaternary prevention, avoiding excessive laboratory and imaging tests. This approach contrasts with prenatal care linked to health insurance plans, where tests are frequently requested for low-risk pregnant women that are not part of the MH protocol or the Technical Manual for Birth Centers^(4,10)^.

The excessive performance of tests can contribute to ineligibility, as an altered result, even if the test is not part of the public health prenatal routine or the FBC protocol, can make the woman ineligible. On the other hand, easy access to healthcare resources and multidisciplinary care can help prevent and manage potential complications during pregnancy. These factors should be considered when interpreting the association between having health insurance, ineligibility, and admission for delivery (women with health insurance had a lower chance of eligibility but a higher chance of admission for delivery).

In Brazil, the active inclusion and participation of fathers or partners in pregnancy and childbirth care represent a strategy for conscious fatherhood^(21)^, consistent with the finding that living with a partner favored both eligibility and admission for delivery.

Regarding previous pregnancies, more than half of the women were primigravida. Although the proportion of non-eligible women is lower in this group, the chance of eligibility decreased with each new pregnancy, possibly related to a previous cesarean section in the studied group. On the other hand, each new pregnancy reduced the chance of prenatal care discontinuation.

The main reason for ineligibility for follow-up at Casa Angela was a previous cesarean section. It is likely that many women with a prior cesarean seek the FBC in an attempt to have a vaginal delivery, as these women have low chances of achieving a vaginal birth after cesarean (VBAC), especially in the private healthcare system^(22)^. Concerns regarding VBAC are related to the potential for uterine rupture and its consequences for maternal and neonatal outcomes. However, the literature highlights the safety and benefits of VBAC, noting that it can prevent complications associated with repeat cesareans, such as placenta previa and the need for blood transfusion, among others^(23)^. Despite favorable evidence supporting the safety of VBAC compared to repeat cesareans, the FBC faces challenges within the healthcare system in expanding this type of care. These services require more effective support and protection from the MH and municipal health departments to provide greater flexibility in their protocols.

Other prevalent reasons for ineligibility and prenatal care discontinuation (GDM, GHS, GBS, and post-term pregnancy) are also related to previous reproductive history or current clinical or obstetric complications.

The estimated rate of GDM is 18% among women treated by SUS, making it one of the most common conditions during pregnancy. Complications associated with GDM include cesarean section, preeclampsia, and postpartum diabetes risk for the mother; prematurity, macrosomia, shoulder dystocia, hypoglycemia, and perinatal death for the NB^(6)^. Uncontrolled GDM fully justifies its occurrence as a reason for excluding women from the FBC. It is worth noting that in December 2021, Casa Angela’s protocol was updated, and since then, women with controlled GDM have been followed in prenatal care at Casa, expanding eligibility and admission possibilities for delivery for these women.

The incidence of gestational hypertension varies from 6% to 17% in nulliparous women (who made up the majority at Casa Angela) and from 2% to 4% in multiparous women. GHS is the most common clinical complication and represents the leading cause of maternal morbidity and mortality worldwide, being the second leading cause of maternal death, with an estimated prevalence of 10% to 15%^(24)^. The recommended care protocol for birth centers calls for referral to high-risk prenatal care for all women with blood pressure levels ≥ 160/110 mmHg or > 140/90 mmHg in the presence of preeclampsia signs^(10)^.

In Brazil, the prevalence of positive GBS in pregnant women ranges from 14.1% to 27.6%. The vertical transmission rate is also highly variable (35% to 69%), and approximately 1% to 2% of NB infected by the bacteria will develop perinatal GBS disease. Among NB who develop this disease (1% to 2%), up to 20% may result in death; among survivors, the frequency of sequelae is estimated to be between 15% and 30%^(5,24)^.

In the current Casa Angela protocol, the GBS screening test is routinely requested. The screening of all pregnant women aims to identify those colonized by GBS and initiate intrapartum antibiotic prophylaxis, reducing vertical transmission and, consequently, the lethality of this disease^(5,24)^. The National Institute for Health and Care Excellence (NICE), although not recommending universal screening, advises that antibiotic prophylaxis should be administered if the pregnant woman tests positive or has a history of neonatal GBS infection in a previous pregnancy, as the probability of testing positive in this pregnancy is 50%^(25)^.

The incidence of prolonged pregnancy is difficult to estimate, but it ranges between 0.5% and 10%; in such cases, there is an increased risk of perinatal morbidity and mortality. Some reviews show a 1.48-fold increase in the risk of fetal death in late-term pregnancies^(5)^. Casa Angela considers full-term pregnancy to be between 39 weeks and 0/7 days and 40 weeks and 6/7 days, and late-term from 41 weeks and 0/7 days to 41 weeks and 6/7 days. The most current recommendation for low-risk pregnancies is that labor induction should be proposed for pregnancies beyond 41 weeks^(26)^. In the same document, one of the recommendations is the use of mechanical methods for labor induction in outpatient settings, which could be a practice adopted by FBC, benefiting many women^(26)^.

Considering that most women sought the FBC during the second trimester of pregnancy, the main reasons for ineligibility (grouped as previous reproductive history or current clinical complications) are consistent with the predominant gestational age. Similarly, the reasons for prenatal care discontinuation align with complications identified in the third trimester (grouped as current obstetric or clinical complications).

Finally, it is important to reflect on the profile of the women who participated in this study. Women in situations of greater social vulnerability were those who had fewer opportunities for follow-up at Casa Angela. This scenario reflects a systemic social problem in which women with lower education levels, lower incomes, and of brown and black ethnicities have less access to quality, equitable information and care, contributing to the development of health problems and risk factors that prevent them from receiving care at FBC.

Ideally, the FBC model linked to the SUS should be an option accessible to all low-risk pregnant women, regardless of sociodemographic profile. However, we face many cultural, social, and healthcare system barriers in our country. In this context, birth centers can be an effective SUS strategy to offer vulnerable pregnant women support, empowerment, and a positive experience of pregnancy, childbirth, and motherhood. In short, this is a form of humanized, quality care that all women are entitled to, expanded within a “social model of care”^(3)^.

### Study limitations

The limitations of the study are primarily related to its cross-sectional design, which, while allowing for the identification of associations between variables and the formulation of hypotheses, prevents the establishment of cause-and-effect relationships due to potential confounding factors. The use of secondary data and potential information loss may also lead to information bias.

### Contributions to the field

The findings highlight the need to reflect on the admission criteria for FBC and the standardization of protocols based on evidence and the needs of low-risk pregnant women. These findings also contribute to the enhancement of public policies and guidelines on FBC, with the goal of improving strategies for expanding the model among healthcare professionals, managers, and the general population.

The absence of protocols can hinder evidence-based practice, but overly rigid protocols can negatively impact care focused on the woman’s needs. Additionally, these protocols may be perceived by professionals as a source of pressure and interference with the care they provide^(19)^. Understanding the gestational factors that influence the expectations of FBC users and professionals, as well as maternal and neonatal outcomes, can help refine care protocols to ensure safer deliveries.

For Casa Angela specifically, suggestions were made to improve the information system and enhance data collection on eligibility and prenatal follow-up, including the categorization of reasons for ineligibility and prenatal care discontinuation at Casa Angela.

To expand this model in Brazil, we suggest that FBC be initiatives under the SUS, with more effective integration into the primary care network. There needs to be greater recognition and promotion of this care model, both among healthcare professionals and the population at large. In this way, PHU could serve as the entry point, classifying gestational risk and referring only low-risk pregnant women for follow-up at the FBC. Such a flow could optimize the resources of birth centers, which currently serve a significant number of pregnant women who, as identified in this study, do not meet the criteria for prenatal care or admission for delivery.

## CONCLUSIONS

Sociodemographic factors, along with past and current clinical and obstetric history, influence both eligibility and the discontinuation of prenatal follow-up at FBC. Higher education, higher income, Asian ethnicity, and living with a partner increase the likelihood of eligibility and reduce the likelihood of prenatal care discontinuation. Indigenous ethnicity increases both the likelihood of eligibility and prenatal care discontinuation. Older maternal age, a higher number of pregnancies, and having private health insurance decrease the likelihood of both eligibility and prenatal care discontinuation. However, brown or black skin color reduces the likelihood of eligibility and increases the likelihood of prenatal care discontinuation.

## Data Availability

Not applicable.
