# Supplementary material for: Eligibility and discontinuation of prenatal care in a freestanding birth center: a cross-sectional study
Source: Rev Bras Enferm. 2025 Sep 26;78(2):e20240015. doi: 10.1590/0034-7167-2024-0015 (PMC12470213; doi:10.1590/0034-7167-2024-0015)
Supplement: Translation

# Translation: Elegibilidade e interrupção do acompanhamento pré-natal em centro de parto normal peri-hospitalar: estudo transversal

**DOI:** 10.1590/0034-7167-2024-0015pt

**Authors:** Franciely Schermak, Maria Luiza Gonzalez Riesco

**Affiliations:** I Universidade de São Paulo. São Paulo, São Paulo, Brasil

**Keywords:** Centros de Assistência à, Gravidez e ao Parto, Gravidez, Cuidado Pré-Natal, Enfermagem Obstétrica, Estudos Transversais

## Abstract

**Objetivos:**

analisar os fatores relacionados à elegibilidade e interrupção do acompanhamento pré-natal em um centro de parto normal peri-hospitalar (CPNp).

**Métodos:**

estudo transversal, realizado no CPNp Casa Angela, São Paulo, SP, com 9.954 mulheres cadastradas entre 2020-2022. Realizada análise descritiva, com cálculo da razão de chances.

**Resultados:**

43,6% das mulheres eram elegíveis para o pré-natal e 62,9% tiveram interrupção do acompanhamento. Maior escolaridade, renda elevada, etnia asiática/indígena e residir com companheiro(a) aumentaram a chance de elegibilidade; maior idade materna, número de gestações, cor da pele parda/preta e convênio de saúde diminuíram essa chance. Cor da pele parda/preta e etnia indígena aumentaram a chance de interrupção do pré-natal; maior idade materna, número de gestações, maior escolaridade, renda elevada, etnia asiática, residir com companheiro(a) e convênio de saúde diminuíram essa chance.

**Conclusões:**

fatores sociodemográficos e história clínica e obstétrica influenciam a inelegibilidade e interrupção do acompanhamento pré-natal em CPNp.

## INTRODUÇÃO

Os Centros de Parto Normal (CPN) peri-hospitalares (CPNp) e intra-hospitalares (CPNi) são uma estratégia para oferecer uma assistência humanizada, baseada em evidências científicas, e contribuir para a redução das taxas de cesariana^(1)^. Desde 1999, o Ministério da Saúde (MS) brasileiro vem estabelecendo legislação específica e diretrizes para a criação e o funcionamento dos CPN^(2)^. De acordo com a Portaria GM n.º 11/2015, o CPNp deve estar localizado nas imediações do hospital de referência, a uma distância que possa ser percorrida em menos de 20 minutos, com unidades de transporte adequadas. O centro deve garantir a transferência da mulher e do recém-nascido (RN) para o hospital em casos eventuais de risco ou intercorrências, em todos os dias da semana, 24 horas por dia; deve contar com serviços de apoio como referência e garantir a permanência da mulher e do RN no quarto PPP (pré-parto, parto e pós-parto) desde a admissão até a alta^(1)^.

Também denominado “casa de parto”, oferece um “modelo social de cuidados”. Em contraste com o modelo hospitalar típico, o CPNp reconhece o nascimento como uma importante transição de vida para mulheres e famílias, vinculado a experiências positivas no contexto da maternidade e paternidade. Possui características de ambiência e equipe profissional que são eficazes na redução de intervenções e na promoção do bem-estar mental e social dos usuários^(3)^.

Esse equipamento de saúde deve estar inserido na rede de atenção primária, atendendo as usuárias da área de abrangência. Presta assistência ao parto normal e é destinado a mulheres de risco habitual. As enfermeiras obstetras e obstetrizes são responsáveis pelo cuidado integral, contando com o apoio de outros profissionais de enfermagem, podendo haver uma equipe multidisciplinar^(1)^.

Durante o pré-natal, as gestantes devem ser informadas sobre as opções de local para o parto e, estando dentro dos critérios de risco habitual, têm o direito de escolher onde desejam ter seu bebê, recebendo informações e assistência de qualidade. De acordo com as diretrizes nacionais de assistência ao parto normal do MS, “Gestantes de risco habitual que optarem pelo parto em Centro de Parto Normal (extra, peri ou intra-hospitalar), se disponível na sua área de abrangência ou em locais próximos, devem ser apoiadas em sua decisão”^(4)^.

A gravidez é um fenômeno fisiológico e deve ser considerada pelas gestantes e equipes de saúde como parte de uma experiência de vida saudável, que envolve mudanças físicas, sociais e emocionais. No entanto, algumas gestantes podem apresentar maior probabilidade de evolução desfavorável devido a fatores de risco; nesse caso, são denominadas “gestantes de alto risco”. Quando a morbidade e a mortalidade materna e perinatal apresentam proporções iguais ou menores que as da população em geral, a gestação é considerada de risco habitual, sem a necessidade de utilizar alta densidade tecnológica em saúde^(4)^.

Os fatores de risco gestacional podem ser prontamente identificados durante a assistência pré-natal, por meio da anamnese, exame físico geral e obstétrico, e exames complementares. A presença de um ou mais desses fatores não implica a necessidade imediata de recursos propedêuticos com tecnologia mais avançada do que aqueles comumente oferecidos no pré-natal de risco habitual. No entanto, esses fatores indicam que a equipe de saúde deve ter uma atenção redobrada com essas gestantes. Quando são identificados fatores associados a um pior prognóstico, pode ser necessário o uso de procedimentos com maior densidade tecnológica, como exames laboratoriais ou de imagem especializados, internação hospitalar e monitoramento de malformações fetais^(4)^.

Conforme a literatura, nem sempre a gestante precisa ser encaminhada ao pré-natal de alto risco; muitas situações podem ser resolvidas nos serviços de atenção primária à saúde, e casos de risco real ou situações evitáveis devem ser referenciados. A gestante pode retornar ao nível primário de atenção quando a situação for resolvida. Cerca de 10% a 15% das gestantes representam o grupo que necessita de acompanhamento no alto risco^(5)^.

Os fatores de risco são classificados em três grupos principais, que devem ser identificados e manejados, com indicação de encaminhamento ao alto risco ou a serviços de urgência e emergência. Segundo orientação do MS, esses fatores são classificados como: 1) Fatores de risco que devem ser identificados e cuidados (relacionados às características individuais, às condições sociodemográficas desfavoráveis, à história reprodutiva anterior e à gravidez atual); 2) Fatores de risco que indicam necessidade de encaminhamento ao alto risco (relacionados às condições prévias, à história reprodutiva anterior e à gravidez atual); 3) Fatores que indicam encaminhamento a urgência e emergência^(5)^.

Em 2018, no Reino Unido, por iniciativa da *Midwifery Unit Network* (MUNet) e da *European Midwives Association* (EMA), foi publicado o documento *Midwifery Unit Standard*, com diretrizes para elegibilidade e escolha do local de parto, baseadas em evidências científicas^(6)^.

No Brasil, apesar das políticas existentes, o modelo CPNp ainda é pouco conhecido e raramente disponível. Em 2022, apenas 0,7% dos nascimentos ocorreram em estabelecimentos de saúde não hospitalares^(7)^. Por outro lado, há uma demanda crescente de mulheres que buscam por um parto humanizado fora do contexto hospitalar, como observado no serviço analisado no presente estudo. Ademais, por serem ambientes voltados para mulheres de risco habitual, há um número elevado de transferências para hospitais^(8,9)^, e presume-se também que o número de interrupções do atendimento durante a gestação seja significativo.

Em 2019, a Secretaria Municipal de São Paulo estabeleceu ações para promover a humanização da assistência e qualificar a atenção ao nascimento e parto. Entre essas ações, criou um grupo de trabalho que elaborou o *Manual Técnico das Casas de Parto*, com o objetivo de sistematizar o processo de trabalho e alinhar tecnicamente as demandas e necessidades das parturientes e seus neonatos nas duas casas de parto em funcionamento na cidade. O manual estabelece os critérios para o acompanhamento da gestação das mulheres atendidas nesse ambiente^(10)^.

De acordo com o manual, o acompanhamento da gestação no CPNp é opcional, mas, na admissão para o parto, deve ser verificado se a gestação é de risco habitual, sem fatores que indiquem morbidade materna ou neonatal. Uma avaliação criteriosa deve ser realizada para garantir que o parto também é de risco habitual^(10)^.

O presente estudo foi proposto considerando as barreiras de acesso, a falta de reconhecimento sobre a segurança e o funcionamento dos CPNp, e a escassa literatura sobre o tema.

## OBJETIVOS

Analisar os fatores relacionados à elegibilidade e à interrupção do acompanhamento da mulher durante o pré-natal em um CPNp.

## MÉTODOS

### Aspectos éticos

O estudo foi conduzido de acordo com as diretrizes éticas nacionais e internacionais e aprovado pelo Comitê de Ética em Pesquisa da Escola de Enfermagem da Universidade de São Paulo, cujo parecer está anexado à presente submissão. Não foi necessária a aplicação de termo de consentimento livre e esclarecido, pois se trata de um estudo que utilizou o banco de dados da instituição e relatórios do sistema de prontuário eletrônico, com os dados analisados e apresentados de forma agregada.

### Desenho, período e local do estudo

Trata-se de um estudo transversal, com dados relativos ao período de 2020 a 2022. As fontes de dados foram o cadastro de gestantes, o prontuário eletrônico, relatórios mensais enviados à Secretaria Municipal de Saúde de São Paulo e o banco de dados dos partos. Para o relato deste manuscrito, foram adotadas as recomendações do *STrengthening the Reporting of OBservational studies in Epidemiology* (STROBE).

O estudo foi realizado no CPNp Casa Angela - Centro de Parto Humanizado, localizado na cidade de São Paulo, SP. Trata-se de uma instituição sem fins lucrativos, administrada pela Associação Comunitária Monte Azul, que atende usuárias do Sistema Único de Saúde (SUS). São admitidas para o parto exclusivamente gestantes de risco habitual.

Gestantes em qualquer fase da gravidez realizam um cadastro on-line, com dados de identificação, sociodemográficos, obstétricos, clínicos e os principais motivos de não elegibilidade (cesárea no parto anterior, diabetes, uso de medicação para hipertensão, curetagem até seis meses antes da gravidez atual, gestação múltipla). Após essa etapa, a gestante passa por uma consulta de triagem, realizada on-line. Se confirmada a elegibilidade, é agendada a primeira consulta pré-natal presencial, a partir da 28ª semana de gestação.

O acompanhamento pré-natal e os partos são assistidos por enfermeiras obstétricas e obstetrizes. Quando há algum fator de risco que impossibilita a mulher de ter o parto no CPNp, o pré-natal é interrompido, e a gestante recebe orientações sobre o motivo e um encaminhamento para a unidade básica de saúde (UBS) onde está realizando o pré-natal. A inserção na rede municipal viabiliza a formalização dos fluxos de referência e contrarreferência com as UBS. Quando há necessidade de atendimento de urgência, a gestante é encaminhada ao hospital de referência ou ao hospital de escolha da mulher, quando ela possui convênio de saúde.

O acompanhamento gestacional ocorre em consultas individuais e coletivas (realizadas em grupo, com gestantes na mesma idade gestacional), intercaladas com a UBS, seguindo a periodicidade recomendada pelo MS. Em todas as consultas de pré-natal, os fatores de risco são reavaliados e, se em algum momento ocorrer intercorrência que impossibilite o parto no CPNp, o acompanhamento é interrompido.

A admissão pode ocorrer a partir de 37 semanas de gestação e até 41 semanas e 0/7 dias. Quando há necessidade de transferência materna ou neonatal para o hospital, a remoção é realizada por ambulância da própria instituição para o hospital de referência, localizado nas imediações do CPNp.

### População e critérios de inclusão e exclusão

A população do estudo foi composta por todas as mulheres as mulheres gestantes que fizeram o cadastro para o acompanhamento pré-natal e o parto, durante o período de 1 de janeiro de 2020 a 31 de dezembro de 2022. O critério de inclusão foi ter preenchido, integralmente, o cadastro para iniciar o acompanhamento, independentemente de ter ou não iniciado o pré-natal no CPNp. Foram excluídas as mulheres que preencheram o cadastro, porém no banco de dados da instituição não havia informação de elegibilidade e tampouco informações sobre acompanhamento no CPNp.

### Exposição e desfechos

Foram considerados como exposição: idade materna; número de gestações; escolaridade; renda familiar; cor da pele/etnia; residência com companheiro; convênio médico.

Os desfechos foram a elegibilidade para o acompanhamento pré-natal na Casa Angela e a interrupção do pré-natal entre as mulheres não admitidas para o parto no CPNp.

### Análise dos resultados e estatística

Os dados foram organizados e categorizados em planilha *Google Sheets*, importados para planilhas Excel e analisados pelo programa R versão 4.3.2.

Para a análise descritiva das variáveis contínuas, foram calculados a média, desvio padrão (d.p.), mediana, valores mínimo e máximo, quartil inferior (Q1) e quartil superior (Q3). Para as variáveis categóricas, foram calculadas frequências absoluta e relativa.

Para análise da relação entre as variáveis de exposição e os desfechos de elegibilidade, interrupção e admissão para o parto foi calculada a razão de chance (OR).

## RESULTADOS

No período de janeiro de 2020 a dezembro de 2022, 10.958 mulheres realizaram o cadastro para iniciarem o acompanhamento pré-natal e o parto no CPNp; destas, 1.004 (9,2%) foram excluídas porque a informação sobre a elegibilidade e o seguimento do acompanhamento na Casa Angela não estava disponível nas fontes de dados utilizadas no estudo.

A população foi constituída por 9.954 mulheres que preencheram o cadastro, com informações sobre condições de elegibilidade para o atendimento no CPNp. De acordo com os critérios da triagem *on-line*, 5.619 (56,4%) não atenderam ao protocolo para ingressar no pré-natal e 4.335 (43,6%) foram consideradas elegíveis ao acompanhamento na Casa Angela. Dentre as mulheres elegíveis, 1.608 foram admitidas para o parto e 2.727 tiveram o atendimento interrompido, imputando a taxa de descontinuidade (taxa de insucesso) de 62,9% (Figura 1).

**Figura 1:** Fluxograma das mulheres cadastradas e atendidas, São Paulo, São Paulo, Brasil, 2020-2022

As Tabelas 1 e 2 mostram as características sociodemográficas e obstétricas da população.

**Tabela 1:** Distribuição das mulheres elegíveis e com interrupção do pré-natal segundo as características sociodemográficas, São Paulo, São Paulo, Brasil, 2020-2022

Variável	Elegível		Total	Interrupção do pré-natal	Admissão para o parto	Total
	Sim	Não				
	n=4.335 (%)	n=5.619 (%)	N=9.954 (%)	n=2.727	n=1.608	N=4.335
Cor da pele/etnia	4.112	4.861	8.973	2.645	1.467	4.112
Branca	1.923 (46,7)	2.191 (53,2)	4.113 (45,8)	1.185 (61,6)	738 (38,4)	1.923 (46,9)
Parda	1.469 (44,9)	1.804 (55,1)	3.273 (36,5)	975 (66,4)	494 (33,6)	1.469 (35,7)
Preta	606 (44,9)	745 (55,1)	1.351 (15,1)	413 (68,2)	193 (31,8)	606 (14,7)
Amarela	84 (46,9)	95 (53,1)	179 (2,0)	51 (60,7)	33 (39,3)	84 (2,0)
Indígena	30 (52,6)	27 (47,4)	57 (0,6)	21 (70,0)	9 (30,0)	30 (0,7)
Escolaridade	4.109	4.862	8.971	2.643	1.466	4.109
Fundamental incompleto	130 (35,8)	233 (64,2)	363 (4,0)	93 (71,5)	37 (28,5)	130 (3,2)
Fundamental completo	192 (36,0)	342 (64,0)	534 (5,9)	135 (70,3)	57 (29,7)	192 (4,6)
Médio completo	2.193 (45,0)	2.686 (55,0)	4.879 (54,4)	1.423 (64,9)	770 (35,1)	2.193 (53,4)
Superior completo	1.594 (49,9)	1.601 (50,1)	3.195 (35,6)	992 (62,2)	602 (37,8)	1.594 (38,8)
Reside com companheiro(a)	4.213	5.542	9.755	2.718	1.495	4.213
Sim	3.506 (46,2)	4.076 (53,8)	7.582 (77,7)	2.221 (63,3)	1.285 (36,7)	3.506 (83,2)
Não	707 (32,5)	1.466 (67,5)	2.173 (22,3)	497 (70,3)	210 (29,7)	707 (16,8)
Renda familiar (salários-mínimos)	4.112	4.865	8.977	2.645	1.467	4.112
≤ 1	642 (39,7)	976 (60,3)	1.618 (18,0)	440 (68,5)	202 (31,5)	642 (15,6)
2	1.352 (46,8)	1.540 (53,2)	2.892 (32,2)	855 (63,4)	494 (36,6)	1.352 (33,0)
3	898 (47,6)	989 (52,4)	1.887 (21,0)	594 (66,1)	304 (33,9)	898 (21,8)
4	480 (48,3)	512 (57,7)	992 (11,0)	288 (59,9)	192 (40,1)	480 (11,6)
5	302 (52,2)	275 (47,8)	577 (6,4)	190 (62,8)	112 (37,2)	302 (7,3)
6	155 (40,4)	229 (59,6)	384 (4,3)	101 (65,2)	54 (34,8)	155 (3,8)
> 6	283 (45,1)	344 (54,9)	627 (7,0)	174 (61,5)	109 (38,5)	283 (6,9)
Convênio de saúde	4.117	5.063	9.180	2.646	1.471	4.117
Sim	538 (36,2)	948 (63,8)	1.486 (16,2)	307 (57,1)	231 (42,9)	538 (13,1)
Não	3.579 (46,5)	4.115 (53,5)	7.694 (83,8)	2.339 (65,4)	1.240 (34,6)	3.579 (86,9)

**Tabela 2:** Distribuição das mulheres elegíveis e com interrupção do pré-natal segundo a idade e número de gestações, São Paulo, São Paulo, Brasil, 2020-2022

Variável	n	Média (d.p.)	Mediana	Min.-Máx.	Q1-Q3
Elegibilidade					
Idade (anos)					
Elegível	4.332	27,3 (5,4)	27	13-44	23-31
Não elegível	5.611	27,4 (5,8)	27	14-45	23-31
Número de gestações					
Elegível	4.241	1,5 (0,9)	1	1-8	1-2
Não elegível	5.585	1,6 (0,9)	1	1-8	1-2
Interrupção do pré-natal					
Idade (anos)					
Interrupção do pré-natal	2.724	27,3 (5,5)	27	13-44	23-31
Admissão para o parto	1.608	27,4 (5,2)	27	14-42	24-31
Número de gestações					
Interrupção do pré-natal	2.642	1,5 (0,8)	1	1-8	1-2
Admissão para o parto	1.599	1,6 (0,9)	1	1-8	1-2

O perfil das mulheres segundo a elegibilidade para iniciar o pré-natal e a interrupção do acompanhamento indica que aquelas com idade menor que 20 anos e maior ou igual a 40 anos prevaleceram entre as não elegíveis e entre as que sofreram interrupção. A média, mediana e extremos dos anos de idade foi similar entre as elegíveis e não elegíveis e entre aquelas com e sem interrupção.

Em relação à cor da pele ou etnia, mais da metade se declarou parda ou preta, prevalecendo também entre as não elegíveis e entre as que tiveram interrupção do pré-natal.

A maior parte das mulheres tinha ensino médio completo, seguidas por aquelas com ensino superior completo; entre as não elegíveis, prevaleceram aquelas com menor escolaridade (sem ensino médio); no entanto, entre as mulheres com menor escolaridade (com fundamental completo ou incompleto), a interrupção do pré-natal foi proporcionalmente maior, quando comparadas a aquelas com maior escolaridade.

Quanto ao relacionamento, mais de ¾ das mulheres residia com companheiro(a) e a grande maioria respondeu não ter convênio de saúde. A proporção de elegíveis foi maior entre as mulheres que residiam com companheiro(a), assim como entre as que não tiveram interrupção do pré-natal, ou seja, que foram admitidas para o parto. Entre as elegíveis foi maior a proporção de mulheres sem convênio, em contraposição a aquelas admitidas para o parto, prevalecendo as mulheres com convênio.

Em relação à atividade remunerada e à renda familiar, entre as elegíveis prevaleceram as mulheres sem remuneração, renda mensal de 5 salários-mínimos e rendimento ≥ 4 salários-mínimos. Entre as mulheres com pré-natal interrompido, prevaleceram aquelas com remuneração, renda mensal ≤ 1 salário-mínimo e rendimento ≤ 3 salários-mínimos.

No que se refere ao conhecimento sobre a Casa Angela, a maioria das mulheres soube por intermédio de amigos ou familiares e apenas uma pequena proporção foi por indicação de profissional de saúde. Chama também a atenção a prevalência de mulheres não elegíveis que conheceram a Casa pelas redes sociais, coincidindo com aquelas que tiveram interrupção do pré-natal.

Do total de mulheres com acompanhamento anterior na Casa Angela, a maioria foi elegível novamente e entre aquelas com interrupção do acompanhamento pré-natal, a proporção foi maior se a mulher não teve gestação anterior acompanhada na Casa. Tanto a elegibilidade quanto a interrupção do pré-natal foi mais frequente entre as primigestas. Os valores de tendência central e dispersão do número de gestações também foi similar entre as mulheres elegíveis e não elegíveis, bem como entre aquelas com o pré-natal interrompido ou não.

Em relação à idade gestacional na data do cadastro, os grupos estavam, predominantemente, no segundo trimestre da gravidez, com média, mediana e idade mínima menor entre as mulheres não elegíveis e maior entre as admitidas para o parto. Das 2.727 mulheres que tiveram interrupção do acompanhamento pré-natal, 242 (8,9%) tinham idade gestacional ≥ 37 semanas (dado não mostrado em tabela).

Do total de mulheres não elegíveis e de mulheres com o acompanhamento pré-natal interrompido, para apenas 1.363 (24,3%) e 1.466 (53,8%) delas, os motivos de não elegibilidade e da interrupção, respectivamente, estavam descritos no banco de dados. Para parte das mulheres, havia até dois motivos para não elegibilidade e até três motivos para interrupção do pré-natal.

O principal motivo de não elegibilidade das mulheres para ingressarem no acompanhamento da Casa Angela foi a cesariana no parto anterior, representando 37,6% dos motivos, seguido por diabetes mellitus gestacional (DMG) (11,0%) e síndrome hipertensiva gestacional (SHG) (9,8%). Por sua vez, o principal motivo de interrupção do acompanhamento pré-natal foi a SHG, representando 15,6% dos motivos, seguido por resultado positivo para pesquisa de estreptococo do grupo B (EGB) no canal vaginal ou ânus e pós-datismo (dados não mostrados em tabelas).

Os motivos de não elegibilidade e de interrupção foram reagrupados e classificados, conforme apresentado na Tabela 3. Entre os motivos de não elegibilidade, forma mais frequentes aqueles relacionados com a história reprodutiva anterior, seguidos por intercorrência clínica atual e condição clínica prévia à gestação. Em relação à interrupção, forma mais frequentes aqueles relacionados as intercorrências obstétricas e clínicas atuais.

**Tabela 3:** Distribuição das mulheres segundo o motivo da inelegibilidade e da interrupção do pré-natal, São Paulo, São Paulo, Brasil, 2020-2022

Variável	n	%
Motivo da inelegibilidade	1.534	100
História reprodutiva anterior (cesariana ou curetagem)	614	39,9
Intercorrência clínica atual^*^	487	31,8
Condições clínicas prévias à gestação atual†	196	12,8
Intercorrência obstétrica atual‡	99	6,5
Condições individuais e sociodemográficas atuais§	90	5,9
Outros¶	48	3,1
Motivo da interrupção do pré-natal	1.593	100
Intercorrência obstétrica atual‡	826	51,8
Intercorrência clínica atual^*^	628	39,4
Condições individuais e sociodemográficas atuais§	75	4,7
Condições clínicas prévias à gestação atual†	52	3,3
Outros¶	12	0,8

* *Mais frequentes (>1%): síndrome hipertensiva gestacional ou pico hipertensivo, streptococcus B positivo, diabetes mellitus gestacional, infecção do trato urinário, sífilis ativa ou tratada na gestação, anemia; †Mais frequentes (>1%): mioma, obesidade grau 3, doenças da tireoide, asma, transtorno de ansiedade e depressão; ‡Mais frequentes (>1%): pós-datismo >41 semanas, percentil fetal extremo, alterações no volume do líquido amniótico, alteração em cardiotocografia, ultrassom fetal ou doppler, apresentação pélvica, trabalho de parto prematuro, mecônio, rotura prematura das membranas ovulares, altura uterina aumentada, restrição de crescimento intrauterino, gestação múltipla; ¶Situações que aconteceram apenas uma vez ou o motivo não ficou claro nas anotações das profissionais.*

A Tabela 4 apresenta a razão de chances (OR) para elegibilidade e para a interrupção do acompanhamento pré-natal na Casa Angela, calculada para as variáveis de exposição. Os resultados para a elegibilidade são: cada ano da idade materna diminui a chance em 0,3%); cada gestação diminui a chance em 15,6%; cada faixa de escolaridade aumenta a chance em 10%; cada faixa de renda aumenta a chance em 3%;comparadas às brancas, as mulheres pardas têm 7,3% menor chance, as mulheres pretas têm 7,4% menor chance, as amarelas (etnia asiática) têm 0,7% maior chance, as de etnia indígena têm 26,5% maior chance; residir com companheiro aumenta a chance em 78,4%; ter convênio de saúde diminui a chance de ser elegível em 34,7%. Os resultados para a interrupção do acompanhamento pré-natal são: cada ano da idade materna diminui a chance em 0,5%; cada gestação diminui a chance em 10,0%; cada faixa de escolaridade diminui a chance em 7,5%; cada faixa de renda diminui a chance em 3,7%; comparadas às brancas, as mulheres pardas têm 22,9% maior chance, as pretas têm 33,3% maior chance; as mulheres amarelas (etnia asiática) têm 3,7% menor chance, as mulheres de etnia indígena têm 45,3% maior chance; residir com companheiro diminui a chance em 27,0%; ter convênio de saúde diminui a chance 29,5%.

**Tabela 4:** Razão de chance (OR) de elegibilidade para acompanhamento e interrupção do pré-natal, São Paulo, São Paulo, Brasil, 2020-2022

Desfecho	OR
Elegibilidade	
Idade materna	0,997
Número de gestações	0,844
Escolaridade	1,100
Renda familiar	1,030
Cor da pele/etnia	
Branca	1,000
Parda	0,927
Preta	0,926
Amarela	1,007
Indígena	1,265
Residência com companheiro	1,784
Convênio médico	0,653
Interrupção do pré-natal	
Idade materna	0,995
Número de gestações	0,900
Escolaridade	0,925
Renda familiar	0,963
Cor da pele/etnia	
Branca	1,000
Parda	1,229
Preta	1,333
Amarela	0,963
Indígena	1,453
Residência com companheiro	0,730
Convênio médico	0,705

## DISCUSSÃO

Embora possam se configurar como restrições ao acesso, os critérios de elegibilidade para o acompanhamento das gestantes são medidas para minimizar riscos de desfechos maternos e neonatais desfavoráveis, viabilizando o funcionamento dos CPNp. O impacto desses critérios na triagem pré-natal e na admissão de mulheres para o parto se reflete nas taxas de insucesso, dado que mais da metade das gestantes que buscaram o CPNp não era elegível, seja devido a condições prévias ou intercorrências ao longo da gestação.

Existem fatores associados ao risco gestacional que, inicialmente, não justificam o encaminhamento da gestante para atendimento de alto risco, urgência ou emergência^(5)^. O acompanhamento adequado do pré-natal ao pós-parto pressupõe o cuidado contínuo, que favorece a identificação desses fatores. O CPNp, inserido na atenção primária, favorece a integração dos serviços de referência e contrarreferência no atendimento de gestantes, puérperas e RN.

A Casa Angela realiza o acompanhamento pré-natal em parceria com a UBS para manter o vínculo com a rede de atenção primária, que também incentiva as gestantes e os profissionais da rede a conhecerem o modelo de cuidado do CPNp. Vale mencionar que as consultas de pré-natal realizadas na Casa Angela não são registradas nos sistemas de informação da rede municipal. A manutenção das consultas na UBS também possibilita que a atenção primária cumpra os indicadores de número mínimo de consultas.

Os fatores relacionados às características individuais e às condições sociodemográficas desfavoráveis incluem a idade extrema. Gestantes adolescentes são mais propensas a desenvolver complicações como SHG e parto prematuro^(5,11-13)^. Na Casa Angela, as gestantes adolescentes são acolhidas em um projeto dedicado a esse grupo etário, que inclui o início do pré-natal a qualquer momento, antes de 28 semanas (diferentemente do protocolo de início do acompanhamento das demais gestantes). Mesmo que a gestante apresente fatores de risco, o acompanhamento pré-natal é mantido na Casa, porém o parto é realizado no hospital de referência.

As intercorrências prevalentes na gravidez tardia são DMG, SHG e ruptura prematura de membranas^(14)^. A literatura aponta que mais de 70% das gestantes com idade ≥35 anos tiveram algum tipo de complicação^(14)^ e que mais da metade das mulheres a partir dessa idade tiveram cesariana^(15)^. No entanto, a idade materna não deve ser considerada como fator isolado para complicações clínicas e obstétricas, devendo ser observados fatores relacionados à história de saúde e obstétrica pregressa e atual, assim como as condições socioeconômicas^(14)^. Esses estudos corroboram, em parte, com os resultados deste estudo, em que cada ano de aumento da idade materna diminuiu a chance de elegibilidade e aumentou a chance de interrupção do pré-natal.

Na Casa Angela, mais da metade das mulheres se declarou de cor parda ou preta, semelhante aos dados do Sistema de Informação sobre Nascidos Vivos (SINASC), na região Sudeste, em 2021^(16)^. As mulheres pardas e pretas enfrentam maior dificuldade de acesso à informação e atendimento de qualidade, sofrem mais violência obstétrica e representam 60% dos casos de morte materna^(17)^. O viés de cor da pele e etnia corrobora com os resultados deste estudo, em que, comparadas às mulheres de pele branca, as mulheres pardas e pretas tiveram menor chance de serem elegíveis para o acompanhamento e maior chance de interrupção do pré-natal.

Mulheres em situação de vulnerabilidade socioeconômica - menor escolaridade e baixa renda - enfrentam dificuldades no acesso à atenção obstétrica, o que impacta no aumento de riscos obstétricos evitáveis, devido à baixa qualidade do pré-natal, pouco acesso a exames e outras tecnologias, falta de conhecimento e maior propensão a violências^(18)^. O acesso e a qualidade do cuidado durante a gestação podem ser afetados pela baixa escolaridade e renda^(19)^. O perfil das usuárias do CPNp revelou uma escolaridade elevada (grande maioria com ensino médio ou superior completo), em contraste com os dados do SINASC. Na região Sudeste e no Brasil, em 2022, as mulheres com ensino médio ou superior representaram 26,7% e 23,2%, respectivamente^(20)^.

A menor escolaridade e renda predominaram entre as mulheres inelegíveis e com acompanhamento pré-natal interrompido. A maior escolaridade aumentou a chance de elegibilidade e reduziu a chance de interrupção do pré-natal; por sua vez, a chance de elegibilidade foi diretamente proporcional à elevação da faixa de renda, enquanto a chance de interrupção foi inversamente proporcional à elevação da faixa de renda.

O cuidado individualizado no acompanhamento pré-natal em CPNp pode promover a prevenção quaternária, evitando o excesso de exames laboratoriais e de imagem. Esse acompanhamento contrasta com o pré-natal vinculado a convênios de saúde, onde frequentemente são solicitados exames para gestantes de risco habitual que não estão previstos no protocolo do MS e no Manual Técnico das Casas de Parto^(4,10)^.

A realização de exames em excesso pode contribuir para a inelegibilidade, pois, se a gestante apresentar algum resultado alterado, mesmo que o exame não faça parte da rotina pré-natal da rede pública de saúde ou do protocolo do CPNp, ela pode se tornar inelegível. Por outro lado, a facilidade de acesso a recursos de saúde e ao cuidado multidisciplinar pode prevenir e controlar eventuais intercorrências na gestação. Essas situações devem ser consideradas ao interpretar a associação entre ter convênio de saúde, a inelegibilidade e a admissão para o parto (as mulheres com convênio tiveram menor chance de elegibilidade, mas maior chance de admissão para o parto).

No Brasil, a inclusão e participação ativa dos pais ou parceiros nos cuidados durante a gestação e o parto representam uma estratégia para o exercício consciente da paternidade^(21)^, o que está de acordo com o achado de que residir com companheiro favoreceu tanto a elegibilidade quanto a admissão para o parto.

Em relação às gestações anteriores, mais da metade das mulheres era primigesta. Embora nesse grupo a proporção de inelegíveis seja menor, a chance de ser elegível diminuiu a cada nova gestação, o que pode estar relacionado à ocorrência de cesárea anterior no grupo estudado. Em contrapartida, cada nova gestação reduziu a chance de interrupção do pré-natal.

O principal motivo de inelegibilidade para o acompanhamento na Casa Angela foi a cesárea no último parto. É possível que muitas mulheres com cesariana anterior busquem o CPNp na tentativa de terem um parto normal, pois essas mulheres têm baixas chances de um parto vaginal após cesariana (VBAC, sigla em inglês adotada no Brasil que significa *vaginal birth after cesarean*), principalmente no sistema privado^(22)^. O receio em relação ao VBAC está relacionado à possibilidade de rotura uterina e suas consequências para os desfechos maternos e neonatais. No entanto, a literatura aponta a segurança e os benefícios do VBAC, destacando que ele pode prevenir danos associados à cesariana de repetição, como placenta prévia e necessidade de transfusão sanguínea, entre outros^(23)^. Mesmo com evidências favoráveis à segurança do VBAC quando comparado à cesariana de repetição, o CPNp enfrenta dificuldades no sistema de saúde para ampliar esse tipo de atendimento. Esses serviços precisam de um apoio e proteção mais efetivos por parte do MS e das secretarias municipais de saúde, a fim de flexibilizarem os protocolos nesse aspecto.

Os outros motivos prevalentes para a inelegibilidade e interrupção do pré-natal (DMG, SHG, EGB positivo e pós-datismo) também estão relacionados a antecedentes da história reprodutiva ou a intercorrências clínicas ou obstétricas atuais.

A taxa estimada de DMG é de 18% entre as mulheres atendidas pelo SUS, sendo uma das condições mais comuns na gravidez. Entre as complicações associadas ao DMG destacam-se: cesariana, pré-eclâmpsia e risco de diabetes mellitus no pós-parto para a mãe; prematuridade, macrossomia, distocia de ombro, hipoglicemia e morte perinatal para o RN^(6)^. O DMG não controlado justifica plenamente sua ocorrência como motivo de exclusão das mulheres do CPNp. Vale destacar que, em dezembro de 2021, o protocolo da Casa Angela foi atualizado e, desde então, as gestantes com DMG controlada passaram a ser acompanhadas no pré-natal da Casa, ampliando as possibilidades de elegibilidade e admissão para o parto dessas gestantes.

A incidência de hipertensão gestacional varia de 6% a 17% em nulíparas (que foram a maioria na Casa Angela) e de 2% a 4% em multíparas. A SHG é a intercorrência clínica mais comum e representa a principal causa de morbimortalidade materna no mundo, sendo a segunda principal causa de morte materna, com uma estimativa de 10% a 15%^(24)^. O protocolo assistencial recomendado para as casas de parto prevê o encaminhamento ao pré-natal de alto risco de todas as gestantes com níveis pressóricos ≥ 160/110 mmHg ou > 140/90 mmHg na presença de sinais de pré-eclâmpsia^(10)^.

No Brasil, a prevalência de EGB positivo em gestantes varia de 14,1% a 27,6%. A taxa de transmissão vertical também é bastante variável (35% a 69%) e, aproximadamente, 1% a 2% dos RN contaminados pela bactéria desenvolverão a doença perinatal pelo EGB. Entre os RN que desenvolvem essa doença (1% a 2%), até 20% podem evoluir para óbito; entre os sobreviventes, a frequência de sequelas é estimada entre 15% e 30%^(5,24)^.

No atual protocolo da Casa Angela, a solicitação do exame para pesquisa de EGB faz parte da rotina. O rastreamento de todas as gestantes visa identificar aquelas colonizadas pelo EGB e instituir a antibioticoterapia intraparto, reduzindo a transmissão vertical e, consequentemente, a letalidade dessa doença^(5,24)^. O *National Institute for Health and Clinical Excellence* (NICE), mesmo não recomendando o rastreamento universal, indica que a profilaxia com antibiótico seja realizada se a gestante tiver um resultado positivo ou histórico de infecção neonatal por EGB em gestação anterior, pois a probabilidade de a mulher ter resultado positivo nesta gravidez é de 50%^(25)^.

A incidência de gravidez prolongada é difícil de ser estimada, mas varia entre 0,5% e 10%; nesses casos, há um risco aumentado de morbidade e mortalidade perinatal. Algumas revisões mostram um aumento do risco de óbito fetal em 1,48 vezes na gestação a termo tardio^(5)^. A Casa Angela considera como gestação a termo completo aquela que vai de 39 semanas e 0/7 dias a 40 semanas e 6/7 dias, e a termo tardio, de 41 semanas e 0/7 dias a 41 semanas e 6/7 dias. A recomendação mais atual para gestações de risco habitual é que a indução do parto seja proposta para gestantes acima de 41 semanas^(26)^. Nesse mesmo documento, uma das recomendações é o uso de métodos mecânicos para indução do parto e a utilização de ambientes ambulatoriais para esses métodos. Essa pode ser uma prática utilizada em CPNp, que beneficiaria muitas mulheres^(26)^.

Considerando que a procura pelo CPNp ocorreu, com maior frequência, por mulheres no segundo trimestre da gestação, os principais motivos de inelegibilidade (agrupados como história reprodutiva anterior ou intercorrência clínica atual) são compatíveis com a idade gestacional predominante. Da mesma forma, os motivos de interrupção do acompanhamento pré-natal estão condizentes com as intercorrências identificadas no terceiro trimestre da gestação (agrupadas como intercorrência obstétrica ou clínica atual).

Por fim, é importante refletir sobre o perfil das mulheres que participaram deste estudo. As mulheres em situação de maior vulnerabilidade social foram as que tiveram menos oportunidades de acompanhamento na Casa Angela. Esse cenário reflete um problema social e sistêmico, em que mulheres com menor escolaridade, renda mais baixa, e de etnias parda e preta têm menos acesso a informações e cuidados de qualidade e equitativos, contribuindo para o desenvolvimento de problemas de saúde e fatores de risco que as impedem de ser atendidas em CPNp.

Idealmente, o modelo de CPNp vinculado ao SUS deveria ser uma opção acessível a todas as gestantes de risco habitual, independentemente do perfil sociodemográfico. No entanto, enfrentamos muitas barreiras culturais, sociais e do sistema de saúde em nosso país. Nesse contexto, as casas de parto podem ser uma estratégia eficaz do SUS para oferecer às gestantes mais vulneráveis acolhimento, empoderamento e uma vivência positiva da gestação, parto e maternidade. Em suma, trata-se de um cuidado humanizado e de qualidade, ao qual todas têm direito, ampliado em um “modelo social de cuidados”^(3)^.

### Limitações do estudo

As limitações do estudo estão principalmente relacionadas ao desenho transversal, que, embora permita identificar associações entre as variáveis e levantar hipóteses, a possível confusão entre elas impede o estabelecimento de relações de causa e efeito. O uso de dados secundários e as perdas de informações podem gerar viés de informação.

### Contribuições para a área

Os achados sugerem a necessidade de reflexão sobre os critérios de admissão em CPNp e a padronização de protocolos baseados em evidências e nas necessidades das gestantes de risco habitual. Esses achados também contribuem para o aprimoramento de políticas públicas e diretrizes sobre CPNp, com o objetivo de melhorar as estratégias de expansão do modelo por profissionais de saúde, gestores e população.

A ausência de protocolos pode dificultar a prática baseada em evidências, mas protocolos excessivamente rígidos podem prejudicar o cuidado centrado nas necessidades da mulher. Além disso, esses protocolos podem ser percebidos pelos(as) profissionais como uma fonte de pressão e interferência no cuidado que oferecem^(19)^. Conhecer os fatores gestacionais que influenciam as expectativas das usuárias e dos profissionais de CPNp, bem como os desfechos maternos e neonatais, pode aprimorar os protocolos assistenciais, visando à segurança do parto.

Para a Casa Angela, em particular, foram feitas sugestões para aprimorar o sistema de informação e melhorar a obtenção de dados sobre elegibilidade e acompanhamento pré-natal, com a categorização dos motivos de inelegibilidade e de interrupção do pré-natal na Casa.

Para expandir esse modelo no Brasil, sugerimos que os CPNp sejam iniciativas do SUS, com uma integração mais efetiva com a rede de atenção primária. É necessário um trabalho de valorização e incentivo desse modelo assistencial, tanto entre os profissionais de saúde quanto junto à população. Dessa forma, a UBS poderia atuar como porta de entrada, classificando o risco gestacional e direcionando para o acompanhamento no CPNp apenas as gestantes de risco habitual. Esse fluxo poderia otimizar os recursos das casas de parto, que atualmente atendem a uma demanda significativa de gestantes que, conforme identificado no presente estudo, não atendem aos critérios para acompanhamento pré-natal e admissão para o parto.

## CONCLUSÕES

Fatores sociodemográficos, bem como a história clínica e obstétrica pregressa e atual, influenciam tanto a elegibilidade quanto a interrupção do acompanhamento pré-natal em CPNp. Maior escolaridade, maior renda, etnia asiática e residir com o(a) companheiro(a) aumentam a chance de elegibilidade e diminuem a chance de interrupção do pré-natal. A etnia indígena aumenta tanto a chance de elegibilidade quanto a de interrupção do pré-natal. Maior idade materna, maior número de gestações e possuir convênio médico diminuem a chance de elegibilidade e de interrupção do pré-natal. Já a cor da pele parda ou preta reduz a chance de elegibilidade e aumenta a chance de interrupção do pré-natal.

## Data Availability

Não se aplica.
